# Comment on 26 cm Fall Caught on Video Causing Subdural Hemorrhages and Extensive Retinal Hemorrhages in an 8‐Month‐Old Infant

**DOI:** 10.1002/ccr3.9644

**Published:** 2024-12-12

**Authors:** Michelle Shouldice, Jennifer N. Smith

**Affiliations:** ^1^ Department of Pediatrics University of Toronto Toronto Canada; ^2^ The Hospital for Sick Children Toronto Canada

## Abstract

This Letter to the Editor comments on and aims to clarify details regarding the case, 26 cm fall caught on video causing subdural hemorrhages and extensive retinal hemorrhages in an 8‐month‐old infant. The findings in this case are rarely reported in accidental injury events and are, therefore, important to understand.

We read with interest the case report by Brook et al. of an 8‐month‐old infant with subdural and retinal hemorrhages following a fall from a couch with inertial rotational forces and possible previous head injury [1]. We thank the authors for the details provided, which are important in considering the differential diagnosis in a particular case, including the possibilities of accidental and inflicted head trauma, and any possible contributing factors. The existing literature, including systematic reviews from Canada, Australia, and the United Kingdom, supports the predominant opinion that subdural hemorrhages and retinal hemorrhages are highly associated with inflicted injury [2, 3, 4, 5, 6]. As a result, this case report is expected to be of interest and importance to others as they assess cases with findings, which may raise concern for inflicted injury. We therefore are requesting further details regarding this case.

The authors appropriately note previous falls and the possibility that these contributed to the symptoms and findings. The pattern of head circumference increase can be helpful clinically in understanding the sequence of events and underlying contributors to bleeding. The authors noted that the head circumference was large at the 99th percentile 1 month prior to presentation to the hospital. It was reported that 1 month prior to presentation, a fall from a trampoline occurred and there was an episode of shoulder and arm twitching. Could the authors clarify the timing of the fall from the trampoline, relative to the timing of the twitching symptoms and the date of the head circumference? Additionally, is the previous pattern of head circumference growth known to the authors?

The authors describe symptoms of vomiting and poor eating 2 days prior to the fall from the couch. We note that these can be symptoms of traumatic head injury, in addition to other possible etiologies, such as common viral illnesses. Could the authors clarify the clinical assessment regarding the cause of these symptoms, including history, physical examination, or other testing?

Abnormalities of coagulation are typically considered in the differential diagnosis of infants presenting with bleeding (subdural hemorrhages and retinal hemorrhages). Could the authors clarify whether a bleeding disorder work‐up was completed and if so, it would be helpful to understand which tests were completed and the results?

We are interested in the details of the head imaging. Focal subdural hemorrhages may be more typical to result from an accidental household fall, whereas multi‐compartment hemorrhages could suggest more significant injury or alternate mechanism [6]. Were the subdural hemorrhages all supratentorial, or were there also infratentorial hemorrhages? Would it be possible to share an axial or coronal MRI FLAIR image, which may provide improved visualization of the subdural and subarachnoid spaces?

We note the ophthalmology comment that the optic nerves in each eye were mildly swollen, suggesting a possible increase in intracranial pressure. Was any intervention required for increased intracranial pressure? Was there an indication of cerebral edema on the CT scan completed on the day of the described event? It would also be helpful to understand the clinical course of symptoms following the day of the fall from the couch and during hospitalization.

Long‐term follow‐up can likewise be informative. In the months following the injury, what was the child's neurodevelopmental status and follow‐up head imaging?

Case reports of this nature are critical to deepening our understanding of potential mechanisms for subdural hemorrhages and retinal hemorrhages in infants. We read this case report with interest and appreciate the authors' efforts in responding to our questions.

## Author Contributions

**Michelle Shouldice:** conceptualization, data curation, writing – original draft, writing – review and editing. **Jennifer N. Smith:** conceptualization, data curation, writing – original draft, writing – review and editing.

## Conflicts of Interest

The authors declare no conflicts of interest.

## References

[1] C. Brook , W. Squier , and J. Mack , “26 Cm Fall Caught on Video Causing Subdural Hemorrhages and Extensive Retinal Hemorrhages in an 8‐Month‐Old Infant,” Clin Case Reports 12, no. 7 (2024): e9105. 10.1002/ccr3.9105 PMC11199187 38933710

[2] G. Bhardwaj , V. Chowdhury , M. B. Jacobs , K. T. Moran , F. J. Martin , and M. T. Coroneo , “A Systematic Review of the Diagnostic Accuracy of Ocular Signs in Pediatric Abusive Head Trauma,” Ophthalmology 117, no. 5 (2010): 983–992.e917. 20347153 10.1016/j.ophtha.2009.09.040

[3] S. A. Maguire , P. O. Watts , A. D. Shaw , et al., “Retinal Haemorrhages and Related Findings in Abusive and Non‐Abusive Head Trauma: A Systematic Review,” Eye (Lond) 27, no. 1 (2013): 28–36. 23079748 10.1038/eye.2012.213 PMC3545381

[4] S. J. Piteau , M. G. Ward , N. J. Barrowman , and A. C. Plint , “Clinical and Radiographic Characteristics Associated With Abusive and Nonabusive Head Trauma: A Systematic Review,” Pediatrics 130, no. 2 (2012): 315–323. 22778309 10.1542/peds.2011-1545

[5] S. Maguire , N. Pickerd , D. Farewell , M. Mann , V. Tempest , and A. M. Kemp , “Which Clinical Features Distinguish Inflicted From Non‐Inflicted Brain Injury? A Systematic Review,” Arch Dis Child 94, no. 11 (2009): 860–867. 19531526 10.1136/adc.2008.150110

[6] A. M. Kemp , T. Jaspan , J. Griffiths , et al., “Neuroimaging: What Neuroradiological Features Distinguish Abusive From Non‐Abusive Head Trauma? A Systematic Review,” Arch Dis Child 96, no. 12 (2011): 1103–1112. 21965812 10.1136/archdischild-2011-300630

